# Supplementary material for: Luminescent characteristics and mitochondrial COI barcodes of nine cohabitated Taiwanese fireflies
Source: PeerJ. 2022 Oct 28;10:e14195. doi: 10.7717/peerj.14195 (PMC9620971; doi:10.7717/peerj.14195)
Supplement: Supplemental Information 1 [file peerj-10-14195-s001.pdf]

Supplementals

Luminescent characteristics and mitochondrial COI barcodes of nine cohabitated Taiwanese fireflies

King-Siang Goh[1], Liang-Jong Wang[2], Jing-Han Ni[3], Tzi-Yuan Wang[4]*

[1] Genomic Research Center, Academia Sinica, Nankang, Taipei, Taiwan. E-mail: gohks0505@gmail.com
[2] Forest Protection Division, Taiwan Forestry Research Institute, Taipei, Taiwan. E-mail: josephwang23@tfri.gov.tw
[3] Department of Ecological Humanities, Providence University, Taichung, Taiwan. E-mail: 461507994@qq.com
[4] Biodiversity Research Center, Academia Sinica, Nankang, Taipei, Taiwan. E-mail: tziyuan@gmail.com

*Corresponding Author:
Tzi-Yuan Wang
128 Academia Road, Sec. 2, Nankang, Taipei, 115, Taiwan
Email address: tziyuan@email.com

Table S1. 161 mitochondrial COI sequences used in this study.

Table S2. Luminescent spectrum (λ_{max}) and luminescent intensity (nW/cm^2) of nine cohabitated species from two habitats.

Table S3. Morphological measurements of eight adult fireflies. (TL: body length, PL: pronotum length, PW: pronotum width, EL: front wing length, and EW: front wing width)

Fig. S1. Luminescent spectrum of nine cohabitated fireflies.

Fig. S2. NJ tree

Fig. S3. ML tree

Table S1. 161 mitochondrial COI sequences used in this study.

Genus	Species	Accession number	literature
Abscondita	*anceyi*	MH020192	(Hu & Fu 2018a)
Abscondita	*cerata**	MT534192	this study
Abscondita	*cerata**	MT534199	this study
Abscondita	*chinensis*	MK122952	(Wang & Fu 2019)
Abscondita	*chinensis**	BF8	this study
Abscondita	*chinensis**	MT534196	this study
Abscondita	*terminalis*	MK292092	(Chen et al. 2019)
Aquatica	*ficta*	NC035060	(Wang et al. 2017)
Aquatica	*ficta**	MT534197	this study
Aquatica	*lateralis*	LC306678	(Maeda et al. 2017)
Aquatica	*lateralis*	MK779000	(Kim et al. 2021)
Aquatica	*leii*	KF667531	(Jiao et al. 2015)
Aquatica	*wuhana*	KX758086	(Wang et al. 2017)
Aspisoma	*sp.*	EU009322	(Stanger-Hall et al. 2007)
Aspisoma	*sp.*	MZ394518	(Martin et al. 2021)
Asymmetricata	*circumdata*	MK292113	(Chen et al. 2019)
Asymmetricata	*circumdata*	NC032062	(Luan & Fu 2016)
Bicellonycha	*wickershamorum*	EU009302	(Stanger-Hall et al. 2007)
Bicellonychia	*lividipeis*	NC030060	(Amaral et al. 2016)
Caenia	*amplicornis*	EU009289	(Stanger-Hall et al. 2007)
Curtos	*costipennis*	AB608764	(Oba et al. 2011)
Curtos	*costipennis*	MK609965	Zhang and Fu 2019 Direct submission
Curtos	*fulvocapitalis*	NC058281	(Li et al. 2022)
Curtos	*okinawanus*	AB608765	(Oba et al. 2011)
Curtos	*sauteri**	MT534198	this study
Cyphonocerus	*marginatus*	AB608754	(Oba et al. 2011)
Cyphonocerus	*ruficollis*	AB608755	(Oba et al. 2011)
Cyphonocerus	*sanguineus*	MW365445	(Yuan et al. 2021)
Diaphanes	*citrinus*	MK292103	(Chen et al. 2019)
Diaphanes	*citrinus*	NC051869	(Yang & Fu 2019)
Diaphanes	*mendax*	NC044791	(Chen et al. 2019)
Diaphanes	*nubilus*	NC044787	(Chen et al. 2019)
Diaphanes	*pectinealis*	NC044793	(Chen et al. 2019)
Diaphanes	*sp.*	MK292095	(Chen et al. 2019)
Drilaster	*axillaris*	AB608756	(Oba et al. 2011)
Drilaster	*ohbayashii*	AB608757	(Oba et al. 2011)
Drilaster	*okinawensis*	AB608758	(Oba et al. 2011)

Drilaster	*sp.*	MK292100	(Chen et al. 2019)
Ellychnia	*corrusca*	KM845872	Hebert et al. 2014 Direct Submission
Ellychnia	*corrusca*	KR483038	(Hebert et al. 2016)
Ellychnia	*corrusca*	MG242622	Fallon et al. 2017 Direct Submission
Emeia	*pseudosauteri*	MK292112	(Chen et al. 2019)
Emeia	*pseudosauteri*	MN722654	(Liu & Fu 2020)
Lamprigera	*luquanensis sp. nov.*	MK292091	(Chen et al. 2019)
Lamprigera	*yunnana*	KX758087	(Wang et al. 2017)
Lamprohiza	*splendidula*	KM439947	(Hendrich et al. 2015)
Lamprohiza	*splendidula*	KU919134	Rulik and Ahrens 2016 Direct Submission
Lampyris	*noctiluca*	KJ965576	(Pentinsaari et al. 2014)
Lampyris	*noctiluca*	KM450587	(Hendrich et al. 2015)
Lampyris	*noctiluca*	MN122858	Margaryan 2019 Direct submission
Lucidina	*accensa*	AB608771	(Oba et al. 2011)
Lucidina	*biplagiata*	AB608772	(Oba et al. 2011)
Lucidina	*kotbandia*	FJ462784	Kang et al. 2008 Direct Submission
Lucidina	*sp.*	MK292098	(Chen et al. 2019)
Lucidota	*atra*	KM845124	Hebert et al. 2014 Direct Submission
Lucidota	*atra*	KM847871	Hebert et al. 2014 Direct Submission
Lucidota	*atra*	KR128152	Hebert et al. 2015 Direct Submission
Luciola	*cruciata*	AB608760	(Oba et al. 2011)
Luciola	*cruciata*	AB608783	(Oba et al. 2011)
Luciola	*cruciata*	NC022472	Matsui 2013 Direct submission
Luciola	*curtithorax*	NC038225	(Hu & Fu 2018b)
Luciola	*curtithorax**	MT534191	this study
Luciola	*curtithorax**	MT534193	this study
Luciola	*curtithorax**	MT534195	this study
Luciola	*filiformis yayeyamana*	AB608761	(Oba et al. 2011)
Luciola	*filiformis**	MT534201	this study
Luciola	*italica*	KM448530	(Hendrich et al. 2015)
Luciola	*italica*	KM448734	(Hendrich et al. 2015)
Luciola	*kagiana**	MT534200	this study
Luciola	*papariensis*	MK778992	(Kim et al. 2021)
Luciola	*papariensis*	MK778999	(Kim et al. 2021)
Luciola	*parvula*	AB608763	(Oba et al. 2011)
Luciola	*parvula*	LC222417	Oba et al. 2017 Direct submission
Luciola	*singapura*	MW620432	(Jusoh et al. 2021)
Luciola	*sp.*	EU009318	(Stanger-Hall et al. 2007)
Luciola	*sp.*	MK292105	(Chen et al. 2019)

Luciola	*sp.*	MK292108	(Chen et al. 2019)
Luciola(Hotaria)	*unmunsana*	MK778938	(Kim et al. 2021)
Luciola(Hotaria)	*unmunsana*	MK778977	(Kim et al. 2021)
Luciola(Hotaria)	*unmunsana*	NC050947	(Kim et al. 2020)
Micronaspis	*floridana*	EU009314	(Stanger-Hall et al. 2007)
Microphotus	*angustus*	EU009301	(Stanger-Hall et al. 2007)
Phausis	*reticulata*	EU009311	(Stanger-Hall et al. 2007)
Phausis	*rhombica*	KM850440	Hebert et al. 2014 Direct Submission
Phausis	*rhombica*	KR914931	(Hebert et al. 2016)
Phosphaenus	*hemipterus*	KM452081	(Hendrich et al. 2015)
Phosphaenus	*hemipterus*	MW259939	Koehler et al. 2020 Direct submission
Photinus	*australis*	EU009298	(Stanger-Hall et al. 2007)
Photinus	*floridanus*	EU009306	(Stanger-Hall et al. 2007)
Photinus	*ignitus*	KR483936	(Hebert et al. 2016)
Photinus	*ignitus*	KR490880	(Hebert et al. 2016)
Photinus	*indictus*	MG054195	Dewaard 2017 Direct Submission
Photinus	*marginellus*	JF888957	International Barcode of Life (iBOL) Direct Submission
Photinus	*marginellus*	KM845360	Hebert et al. 2014 Direct Submission
Photinus	*marginellus*	KM848322	Hebert et al. 2014 Direct Submission
Photinus	*punctulatus*	EU009312	(Stanger-Hall et al. 2007)
Photinus	*pyralis*	HM433332	International Barcode of Life (iBOL) Direct Submission
Photinus	*pyralis*	KY778696	(Fallon et al. 2018)
Photinus	*tanytoxis*	EU009315	(Stanger-Hall et al. 2007)
Photuris	*aff lucicrescens*	KR490457	(Hebert et al. 2016)
Photuris	*lucicrescens*	EU009290	(Stanger-Hall et al. 2007)
Photuris	*pensylvanica*	AY165656	(Hebert et al. 2003)
Photuris	*pensylvanica*	KM844740	Hebert et al. 2014 Direct Submission
Photuris	*pensylvanica*	MF637320	Dewaard 2017 Direct Submission
Photuris	*quadrifulgens*	HM433520	International Barcode of Life (iBOL) Direct Submission
Photuris	*quadrifulgens*	KJ166508	Dewaard et al. 2014 Direct Submission
Photuris	*quadrifulgens*	KM845417	Hebert et al. 2014 Direct Submission
Photuris	*tremulans*	EU009308	(Stanger-Hall et al. 2007)
Photuris	*tremulans*	KR480659	(Hebert et al. 2016)
Pleotomodes	*needhami*	EU009305	(Stanger-Hall et al. 2007)
Pollaclasis	*bifaria*	KM846040	Hebert et al. 2014 Direct Submission
Pollaclassis	*bifaria*	EU009295	(Stanger-Hall et al. 2007)
Pristolycus	*sagulatus*	AB608773	(Oba et al. 2011)
Pristolycus	*sp.*	MK292099	(Chen et al. 2019)
Pteroptyx	*asymmetria*	KY572920	(Jusoh et al. 2014)

Pteroptyx	*asymmetria*	KY572923	(Jusoh et al. 2014)
Pteroptyx	*bearni*	KY572954	(Jusoh et al. 2014)
Pteroptyx	*bearni*	KY572955	(Jusoh et al. 2014)
Pteroptyx	*maipo*	NC036353	(Fan & Fu 2017)
Pteroptyx	*malaccae*	KY572958	(Jusoh et al. 2014)
Pteroptyx	*malaccae*	KY572961	(Jusoh et al. 2014)
Pteroptyx	*tener*	MT140361	(Cheng et al. 2021)
Pteroptyx	*valida*	KY573051	(Jusoh et al. 2014)
Pteroptyx	*valida*	MW620447	(Jusoh et al. 2021)
Pterotus	*obscuripennis*	EU009303	(Stanger-Hall et al. 2007)
Pygoluciola	*qingyu*	MK292093	(Chen et al. 2019)
Pygoluciola	*qingyu*	NC057261	(Liu & Fu 2020)
Pygoluciola	*sp.*	MK292102	(Chen et al. 2019)
Pyractomena	*angulata*	EU009307	(Stanger-Hall et al. 2007)
Pyractomena	*angulata*	HM433326	International Barcode of Life (iBOL) Direct Submission
Pyractomena	*angulata*	KR482095	(Hebert et al. 2016)
Pyractomena	*borealis*	KM842107	Hebert et al. 2014 Direct Submission
Pyractomena	*palustris*	EU009309	(Stanger-Hall et al. 2007)
Pyractomena	*palustris*	KR488871	(Hebert et al. 2016)
Pyrocoelia	*abdominalis*	AB608766	(Oba et al. 2011)
Pyrocoelia	*analis*	OK323960	Guo 2021 Direct submission
Pyrocoelia	*atripennis*	AB608767	(Oba et al. 2011)
Pyrocoelia	*discicollis*	AB608768	(Oba et al. 2011)
Pyrocoelia	*fumosa*	AB608769	(Oba et al. 2011)
Pyrocoelia	*matsumurai*	AB608770	(Oba et al. 2011)
Pyrocoelia	*pectoralis*	KP763467	Fu and Luan 2015 Direct Submission
Pyrocoelia	*praetexta Yunnan*	MK292115	(Chen et al. 2019)
Pyrocoelia	*praetexta**	MT534194	this study
Pyrocoelia	*pygidialis*	MK292097	(Chen et al. 2019)
Pyrocoelia	*rufa*	AF452048	(Bae et al. 2004)
Pyrocoelia	*thibetana*	MK292117	(Chen et al. 2019)
Pyropyga	*decipiens*	EU009300	(Stanger-Hall et al. 2007)
Pyropyga	*nigricans*	EU009294	(Stanger-Hall et al. 2007)
Pyropyga	*nigricans*	HM433335	International Barcode of Life (iBOL) Direct Submission
Pyropyga	*nigricans*	KM847886	Hebert et al. 2014 Direct Submission
Pyropyga	*nigricans*	KR490073	(Hebert et al. 2016)
Rhagophthalmus	*lufengensis*	NC010969	(Li et al. 2007)
Rhagophthalmus	*ohbai*	AB608775	(Oba et al. 2011)
Rhagophthalmus	*ohbai*	NC010964	(Li et al. 2007)

Sclerotia	*aquatilis*	KP763466	Fu and Luan 2015 Direct Submission
Sclerotia	*flavida*	KP763458	Fu and Luan 2015 Direct Submission
Sclerotia	*fui*	KP763465	Fu and Luan 2015 Direct Submission
Sclerotia	*substriata*	NC027176	(Mu et al. 2016)
Stenocladius	*sp.*	MK292101	(Chen et al. 2019)
Stenocladius	*yoshikawai*	AB608759	(Oba et al. 2011)
Vesta	*saturnalis*	NC044788	(Chen et al. 2019)

Table S2. Light spectrum (λ_{max}) and luminescent **intensity (nW/cm^2) of nine cohabitated species from two habitats.**

Species	Sex	Individuals (n)	λ_{max} (nm)	Luminescent intensity (nW/cm^2)	
				Mean	Maximum
A. Nankang, Taipei:					
Abscondita cerata	female	9	562.3 ± 0.4	164.6 ± 40.6	282.2
	male	14	563.2 ± 0.5	333.4 ± 91.3	1065
Aquatica ficta	female	-	-	-	-
	male	1	567	807.0	
Luciola kagiana	female	3	574.3 ± 0.3	NA	NA
	male	2	575.0 ± 0.0	5.4 ± 4.8	10.2
Luciola curtithorax	female	12	566.3 ± 0.4	157.9 ± 30.4	301.3
	male	26	572.5 ± 0.2	356.1 ± 48.0	814.1
Luciola filiformis	female	-	-	-	-
	male	12	567.3 ± 0.2	182.1 ± 31.2	323.8
B. Nanzhuang, Miaoli:					
Abscondita cerata	female	8	561.8 ± 0.8	102.0 ± 18.5	187
	male	14	564.1 ± 0.4	512.4 ± 198.3	2048
Abscondita chinensis	female	3	571.3 ± 0.3	245.7 ± 83.9	329.7
	male	2	572.0 ± 0.0	332.1	332.1
Aquatica ficta	female	5	564.0 ± 0.5	569.4 ± 101.1	850
	male	16	564.3 ± 0.2	508.1 ± 73.3	1102
Luciola kagiana	female	-	-	-	-
	male	1	572	NA	NA
Curtos sauteri	female	5	554.0 ± 0.3	187.7 ± 55.7	349.3
	male	3	552.7 ± 0.9	347.3 ± 95.9	536.7
Curtos costipennis	female	1	554	462	
	male	-	-	-	-
Pyrocoelia praetexta	larva*	3	552.7 ± 0.9	NA	NA

* light spectra were only successfully recorded from larvae.

Table S3. Morphological measurements of eight adult fireflies. (TL: body length, PL: pronotum length, PW: pronotum width, EL: front wing length, and EW: front wing width)

Species	Specimen number	TL (mm)	PL (mm)	PW (mm)	EL (mm)	EW (mm)
Abscondita chinensis						
Female	3	10.49±0.26	2.03±0.1	3.1±0.2	8.12±0.49	3.88±0.10
Male	2	9.58±0.15	2.05±0.04	2.83±0.04	7.4±0.09	3.29±0.02
Total	5	10.12±0.5	2.03±0.08	2.99±0.21	7.83±0.52	3.64±0.30*
Abscondita cerata						
Female	28	10.01±0.7	2.02±0.18	3.14±0.28	7.77±0.56	4.05±0.32
Male	30	9.37±0.32	1.94±0.19	2.76±0.23	7.41±0.36	3.55±0.23
Total	68	9.69±0.63	1.98±0.19***	2.95±0.32	7.58±0.5	3.79±0.37
Aquatica ficta						
Female	5	9.5±1.04	2.03±0.11	3.03±0.34	7.46±0.85	3.67±0.41
Male	15	8.6±0.64	1.79±0.16	2.63±0.26	6.6±0.56	3.18±0.28
Total	20	8.83±0.85	1.85±0.18**	2.73±0.33	6.81±0.74	3.3±0.38
Luciola kagiana						
Female	1	10.52±0	2.08±0	3.06±0	8.65±0	3.85±0
Male	6	9.68±0.63	1.94±0.13	2.89±0.19	7.8±0.46	3.46±0.17
Total	7	9.8±0.65	1.96±0.13	2.91±0.19	7.92±0.52	3.52±0.21
Luciola curtithorax						
Female	2	6.67±0.11	1.28±0.19	2.08±0.03	5.02±0.02	2.61±0.1
Male	3	6.10±0.19	0.94±0.2	1.83±0.18	4.51±0.35	2.15±0.08
Total	5	6.33±0.32*	1.07±0.26	1.93±0.19	4.71±0.37	2.33±0.24
Luciola filiformis						
Female	0					
Male	1	5.93	1.26	1.53	4.63	2.07
Total	1	5.93	1.26	1.53	4.63	2.07
Curtos sauteri						
Female	4	6.43±0.58	1.28±0.2	1.88±0.28	5.07±0.57	2.47±0.19
Male	1	6.13	1.13	1.83	4.87	2.09
Total	5	6.37±0.53	1.25±0.19	1.87±0.26	5.03±0.51	2.39±0.23
Curtos costipennis						
Female	1	7.32	1.68	2.26	5.72	2.54
Male	0					
Total	1	7.32	1.68	2.26	5.72	2.54

* *p-value < 0.05*, ** p-value < 0.01, *** p-value < 0.001

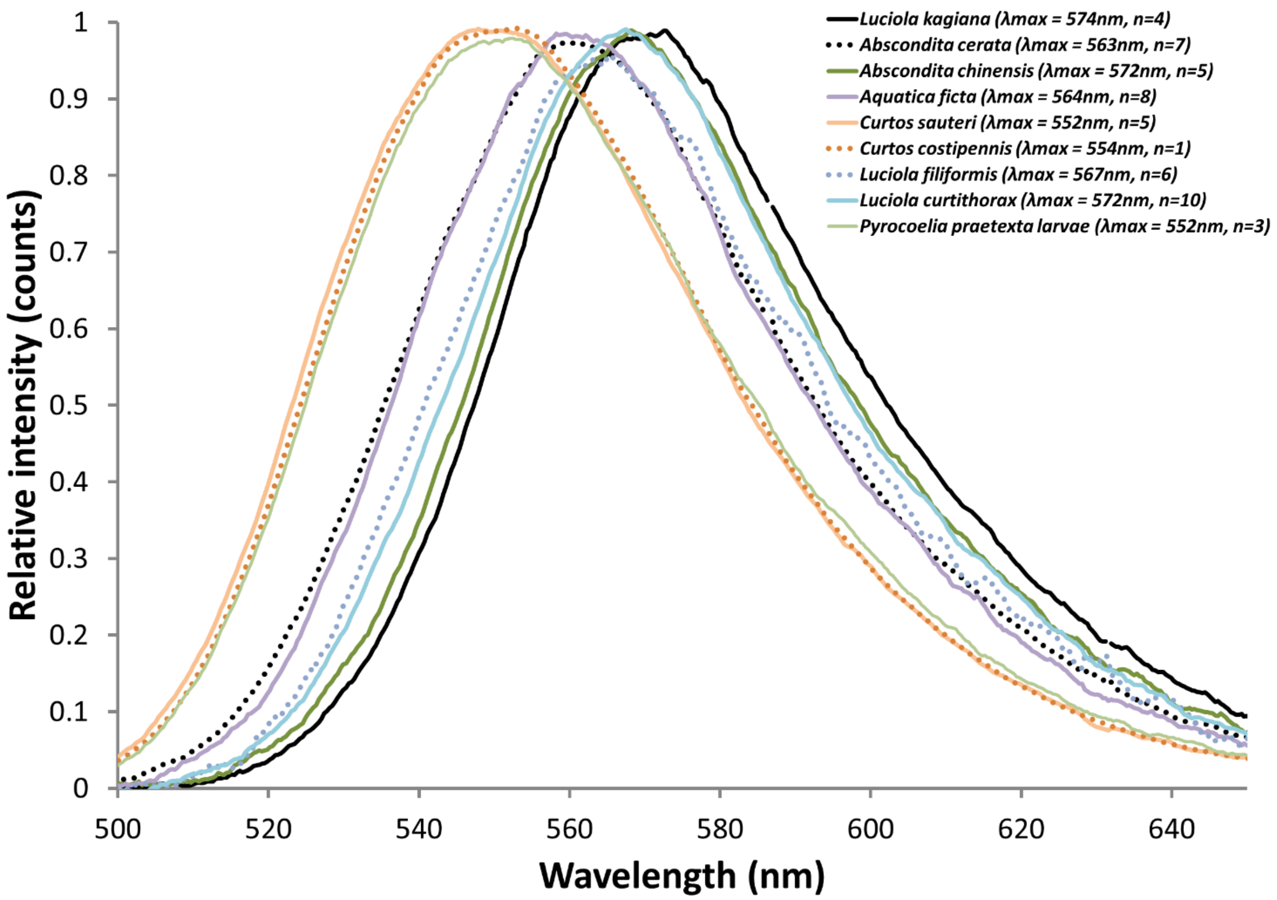

Fig. S1. Luminescent spectrum of nine cohabitated fireflies.

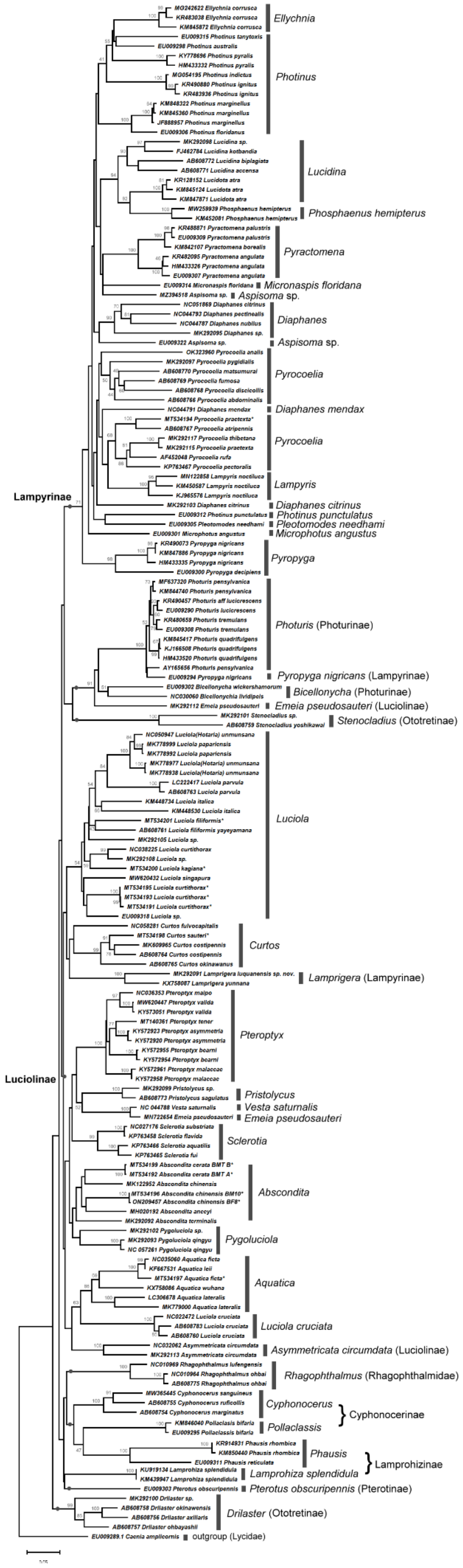

Fig. S2. NJ tree.

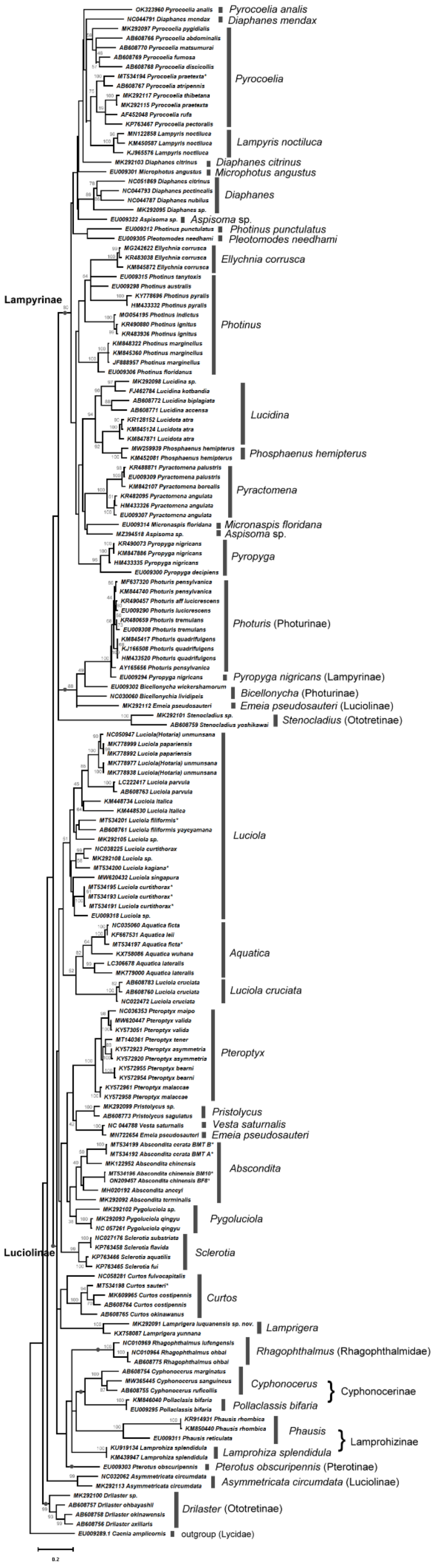

Fig. S3 ML tree

Reference:

Amaral DT, Mitani Y, Ohmiya Y, and Viviani VR. 2016. Organization and comparative analysis of the mitochondrial genomes of bioluminescent Elateroidea (Coleoptera: Polyphaga). *Gene* 586:254-262. 10.1016/j.gene.2016.04.009

Bae JS, Kim I, Sohn HD, and Jin BR. 2004. The mitochondrial genome of the firefly, Pyrocoelia rufa: complete DNA sequence, genome organization, and phylogenetic analysis with other insects. *Molecular Phylogenetics and Evolution* 32:978-985. 10.1016/j.ympev.2004.03.009

Chen X, Dong Z, Liu G, He J, Zhao R, Wang W, Peng Y, and Li X. 2019. Phylogenetic analysis provides insights into the evolution of Asian fireflies and adult bioluminescence. *Molecular Phylogenetics and Evolution* 140:106600. 10.1016/j.ympev.2019.106600

Cheng S, Mat-Isa MN, Sapian IS, and Ishak SF. 2021. The nearly complete mitogenome of the Southeast Asian firefly Pteroptyx tener (Coleoptera: Lampyridae). *Mol Biol Rep* 48:1281-1290. 10.1007/s11033-021-06189-0

Fallon TR, Lower SE, Chang CH, Bessho-Uehara M, Martin GJ, Bewick AJ, Behringer M, Debat HJ, Wong I, Day JC, Suvorov A, Silva CJ, Stanger-Hall KF, Hall DW, Schmitz RJ, Nelson DR, Lewis SM, Shigenobu S, Bybee SM, Larracuente AM, Oba Y, and Weng JK. 2018. Firefly genomes illuminate parallel origins of bioluminescence in beetles. *Elife* 7. ARTN e36495
10.7554/elife.36495

Fan Y, and Fu X. 2017. The complete mitochondrial genome of the firefly, Pteroptyx maipo (Coleoptera: Lampyridae). *Mitochondrial DNA B Resour* 2:795-796. 10.1080/23802359.2017.1398598

Hebert PDN, Cywinska A, Ball SL, and DeWaard JR. 2003. Biological identifications through DNA barcodes. *Proceedings of the Royal Society B-Biological Sciences* 270:313-321. 10.1098/rspb.2002.2218

Hebert PDN, Ratnasingham S, Zakharov EV, Telfer AC, Levesque-Beaudin V, Milton MA, Pedersen S, Jannetta P, and deWaard JR. 2016. Counting animal species with DNA barcodes: Canadian insects. *Philosophical Transactions of the Royal Society B-Biological Sciences* 371. 10.1098/rstb.2015.0333

Hendrich L, Moriniere J, Haszprunar G, Hebert PD, Hausmann A, Kohler F, and Balke M. 2015. A comprehensive DNA barcode database for Central European beetles with a focus on Germany: adding more than 3500 identified species to BOLD. *Mol Ecol Resour* 15:795-818. 10.1111/1755-0998.12354

Hu J, and Fu X. 2018a. The complete mitochondrial genome of the firefly, Abscondita anceyi (Olivier) (Coleoptera: Lampyridae). *Mitochondrial DNA B Resour* 3:442-443. 10.1080/23802359.2018.1456373

Hu J, and Fu X. 2018b. The complete mitochondrial genome of the firefly, Luciola curtithorax (Coleoptera: Lampyridae). *Mitochondrial DNA B Resour* 3:378-379. 10.1080/23802359.2018.1437817

Jiao HW, Ding MH, and Zhao HB. 2015. Sequence and organization of complete mitochondrial genome of the firefly, Aquatica leii (Coleoptera: Lampyridae). *Mitochondrial DNA* 26:775-

776. 10.3109/19401736.2013.855746

Jusoh WFA, Ballantyne L, Chan SH, Wong TW, Yeo DR, Nada B, and Chan KO. 2021. Molecular Systematics of the Firefly Genus Luciola (Coleoptera: Lampyridae: Luciolinae) with the Description of a New Species from Singapore. *Animals* 11. 10.3390/ani11030687

Jusoh WFA, Hashim NR, Saaksjarvi IE, Adam NA, and Wahlberg N. 2014. Species Delineation of Malaysian Mangrove Fireflies (Coleoptera: Lampyridae) Using DNA Barcodes. *Coleopterists Bulletin* 68:703-711. Doi 10.1649/0010-065x-68.4.703

Kim MJ, Park JS, and Kim I. 2020. Complete mitochondrial genome of the Korean endemic firefly, Luciola unmunsana (Coleoptera: Lampyridae). *Mitochondrial DNA B Resour* 5:3165-3167. 10.1080/23802359.2020.1806753

Kim TS, Kwon K, and Jang GS. 2021. Variations in the Distribution and Genetic Relationships among Luciola unmunsana Populations in South Korea. *Land* 10. ARTN 730

10.3390/land10070730

Li W, Liu Q, and Fu X. 2022. The complete mitochondrial genome of the firefly Curtos fulvocapitalis (Coleoptera: Lampyridae). *Mitochondrial DNA B Resour* 7:1-3. 10.1080/23802359.2021.1958080

Li X, Ogoh K, Ohba N, Liang X, and Ohmiya Y. 2007. Mitochondrial genomes of two luminous beetles, Rhagophthalmus lufengensis and R. ohbai (Arthropoda, Insecta, Coleoptera). *Gene* 392:196-205. 10.1016/j.gene.2006.12.017

Liu Q, and Fu X. 2020. The genetic variations in the mitochondrial genomes of three Luciolinae fireflies. *Mitochondrial DNA B Resour* 5:3210-3214. 10.1080/23802359.2020.1806126

Luan X, and Fu X. 2016. The complete mitochondrial genome of the firefly, Asymmetricata circumdata (Motschulsky) (Coleoptera: Lampyridae). *Mitochondrial DNA B Resour* 1:553-555. 10.1080/23802359.2016.1199000

Maeda J, Kato DI, Arima K, Ito Y, Toyoda A, and Noguchi H. 2017. The complete mitochondrial genome sequence and phylogenetic analysis of Luciola lateralis, one of the most famous firefly in Japan (Coleoptera: Lampyridae). *Mitochondrial DNA Part B-Resources* 2:546-547. 10.1080/23802359.2017.1365640

Martin GJ, Lower SE, Suvorov A, and Bybee SM. 2021. Molecular Evolution of Phototransduction Pathway Genes in Nocturnal and Diurnal Fireflies (Coleoptera: Lampyridae). *Insects* 12. ARTN 561

10.3390/insects12060561

Mu FJ, Ao L, Zhao HB, and Wang K. 2016. Characterization of the complete mitochondrial genome of the firefly, Luciola substriata (Coleoptera: Lampyridae). *Mitochondrial DNA A DNA Mapp Seq Anal* 27:3360-3362. 10.3109/19401736.2015.1018221

Oba Y, Branham MA, and Fukatsu T. 2011. The Terrestrial Bioluminescent Animals of Japan. *Zoological Science* 28:771-789. 10.2108/zsj.28.771

Pentinsaari M, Hebert PD, and Mutanen M. 2014. Barcoding beetles: a regional survey of 1872 species reveals high identification success and unusually deep interspecific divergences. *PLoS One* 9:e108651. 10.1371/journal.pone.0108651

Stanger-Hall KF, Lloyd JE, and Hillis DM. 2007. Phylogeny of North American fireflies (Coleoptera : Lampyridae): Implications for the evolution of light signals. *Molecular Phylogenetics and Evolution* 45:33-49. 10.1016/j.ympev.2007.05.013

Wang JL, and Fu XH. 2019. The complete mitochondrial genome of the firefly, Abscondita chinensis (Coleoptera: Lampyridae). *Mitochondrial DNA Part B-Resources* 4:1599-1600. 10.1080/23802359.2018.1551090

Wang K, Hong W, Jiao HW, and Zhao HB. 2017. Transcriptome sequencing and phylogenetic analysis of four species of luminescent beetles. *Scientific Reports* 7. ARTN 1814
10.1038/s41598-017-01835-9

Yang Z, and Fu XH. 2019. The complete mitochondrial genome of the firefly, Diaphanes citrinus (Olivier), (coleoptera: Lampyridae). *Mitochondrial DNA Part B-Resources* 4:2986-2987. 10.1080/23802359.2019.1664351

Yuan L, Ge X, Xie G, Liu H, and Yang Y. 2021. First Complete Mitochondrial Genome of Melyridae (Coleoptera, Cleroidea): Genome Description and Phylogenetic Implications. *Insects* 12. 10.3390/insects12020087
